# Aggression, Violence and Injury in Minor League Ice Hockey: Avenues for Prevention of Injury

**DOI:** 10.1371/journal.pone.0156683

**Published:** 2016-06-03

**Authors:** Michael D. Cusimano, Gabriela Ilie, Sarah J. Mullen, Christopher R. Pauley, Jennifer R. Stulberg, Jane Topolovec-Vranic, Stanley Zhang

**Affiliations:** 1 Division of Neurosurgery, Department of Surgery, Injury Prevention Research Office, Saint Michael’s Hospital, Toronto, Ontario, Canada; 2 Dalhousie University Faculty of Medicine, Department of Community Health and Epidemiology, Halifax, Nova Scotia, Canada; 3 University of Toronto, Family and Community Medicine, Toronto, Ontario, Canada; 4 Faculty of Medicine (Occupational Science and Occupational Therapy), University of Toronto, Toronto, Canada; University of Rome, ITALY

## Abstract

**Background:**

In North America, more than 800,000 youth are registered in organized ice hockey leagues. Despite the many benefits of involvement, young players are at significant risk for injury. Body-checking and aggressive play are associated with high frequency of game-related injury including concussion. We conducted a qualitative study to understand why youth ice hockey players engage in aggressive, injury-prone behaviours on the ice.

**Methods:**

Semi-structured interviews were conducted with 61 minor ice hockey participants, including male and female players, parents, coaches, trainers, managers and a game official. Players were aged 13–15 playing on competitive body checking teams or on non-body checking teams. Interviews were manually transcribed, coded and analyzed for themes relating to aggressive play in minor ice hockey.

**Results:**

Parents, coaches, teammates and the media exert a large influence on player behavior. Aggressive behavior is often reinforced by the player’s social environment and justified by players to demonstrate loyalty to teammates and especially injured teammates by seeking revenge particularly in competitive, body-checking leagues. Among female and male players in non-body checking organizations, aggressive play is not reinforced by the social environment. These findings are discussed within the framework of social identity theory and social learning theory, in order to understand players’ need to seek revenge and how the social environment reinforces aggressive behaviors.

**Conclusion:**

This study provides a better understanding of the players’ motivations and environmental influences around aggressive and violent play which may be conducive to injury. The findings can be used to help design interventions aimed at reducing aggression and related injuries sustained during ice hockey and sports with similar cultures and rules.

## Introduction

For children and adolescents 10–19 years of age, 44% of injuries requiring a visit to the emergency department occur during sports or some other physical activity [1]. Classified as a collision sport, ice hockey (hereafter called simply “hockey”), one of North America’s most popular sports, puts more than 820,000 participants into regular physical activity but also places them at risk for potentially serious injury annually [2, 3]. A report [3] of the Emergency Department Injury Surveillance System found that hockey was the leading sport during which injuries occurred and that 43% of hockey injuries were to the head and neck. Studies have shown that 75–88% of injuries in amateur hockey resulted from collisions, and that at least 25% of these were legal checks [4, 5]. While many players believe legal checks are acceptable in hockey culture, they still account for a significant proportion of injuries in hockey. Aggressive behaviour during hockey results in a number of injuries, ranging in severity from soft-tissue contusions, fractures to concussions, across all age groups [5–15]

The benefits from sports participation for youth are many. Sports have been found to increase physical health, cardiovascular conditioning, strength, and endurance, improve their self-image, decrease the risk of obesity, help youth learn that they can improve their performance and skills through practice and hard work, and teach cooperation and team building skills [16]. Youth that participate in youth team sports learn how to interact with their peers, to assist those who are less skilled, and to learn from those who are more highly skilled. At the same time, we cannot ignore the risks involved in ice hockey and many other contact sports.

In many countries, including the USA and Canada, hockey injuries as a result of aggression in professional and amateur arenas garner extensive media attention [17–19]. Aggression in play has usually been operationalized as purposeful physical, verbal or gestural acts, driven both by competitiveness and an intent to cause physiological or psychological harm [20]. The concept of “injury”, however, can be seen to encompass intrinsic risk factors related to such qualities as bone strength or previous injury history and extrinsic risk factors such as reaction to other athletes, game conditions, officiating decisions or the spectator environment [21] as well as the interrelationships between them [22]. It is qualities such as the latter that were of particular interest in this study. Hockey professionals who engage in aggressive behaviours leading to injury, as well as the media, which disseminates this form of aggression as entertainment [23], both contribute to the socialization of aggressive play. Professional hockey players displaying aggressive play as thus operationally-defined are seen as being poor role models for young viewers [24–26]. An immediate consequence of this type of violence socialization in our culture is that viewers who are now parents have developed the idea that most violence on the ice (e.g., body checking, concussions) may be inconsequential [27] and, while empirical evidence may suggest that parents play many roles, including both positive and negative [28] there is a commonly-held belief that many encourage their children to participate in aggressive behaviours [29]. Their children and adolescents, who are particularly influenced by both media and the way parents respond to live and televised games, become socialized into accepting these high-risk behaviours at a very young age and end up naturally enacting them on ice, rendering themselves vulnerable to injury [2, 4].

Social Learning Theory posits that the socialization journey, from infancy without any culture to the enculturated self, is the result of cultural observation and modelling while growing up [30]. While much of human behaviour is influenced by our genetic makeup, the socialization process can mold it in particular directions by encouraging specific beliefs and attitudes as well as selectively providing experiences. To better understand the factors that influence aggressive and potentially injurious behaviours during hockey among youth, we explored the culture and socialization of youth hockey in a sample of players, parents, coaches, trainers, managers, and a game official in Toronto, Canada. *Culture* can be defined as “the set of shared attitudes, values, goals, and practices that characterizes an institution or organization” [31]. To address this topic, we designed a qualitative study to provide an in-depth perspective on the culture of hockey as seen by players, parents and coaching staff involved in the organization. The first objective of this study was to provide an in-depth analysis of the culture of hockey, specifically with regards to attitudes towards aggression and how it contributes to the frequency of injury. A second objective of the study was to translate the current findings into specific recommendations for the development of preventive interventions in competitive team sports.

## Methods

### Study Design and Sample

Qualitative research is particularly well-suited to exploratory studies for which previous literature is limited. While there are a number of studies that explore attitudes towards aggression in minor hockey players through such means as the use of psychometric tools or player ratings of aggressive incidents on video [32–35] to our knowledge, there are no qualitative studies exploring attitudes towards aggressive play.

We chose a diverse cohort of participants using purposeful sampling from a pool of hockey teams in the Greater Toronto Area, resulting in a final total of 14 teams from a variety of competitive levels of play [36–39]. We also chose to interview a group of “reference others”, including parents, coaches, trainers, and other adults from whom players seek approval and reinforcement [30, 33, 34, 40, 41]. According to Social Learning Theory (SLT) the “reference others” group may play a significant role in shaping players’ attitude and behaviours through observation and modeling [30]. To ensure that interviewee responses were not biased, both offense and defense positions were selected (7 centres, 5 right wing, 6 left wing, 15 defense and 5 goalies). Ten parents, 6 coaches, 4 trainers, 2 managers and a game official were also interviewed. All young players were interviewed face-to-face, 17 of the “reference others” were interviewed in person, and 6 of the “reference others” were interviewed over the phone. We selected a group of early adolescent players because injury often begins to manifest during play at this age, often due to disadvantages related to such variables as height and weight (at this age there is significant variation in young players’ sizes; of those who volunteered their height and weight, they ranged from 160 cm to 178 cm, with weights from 46 kg to 62 kg). This age group also has higher prevalence estimates of injury relative to children or adults [42–46]. The level of play for body checking league teams (competitive) was chosen based on research demonstrating that an increase in concussion frequency is seen with older players and more elite levels of play [47–50] We also had participants from a non-body-checking league (comprised of both females and males) that does not allow body checking, though physical contact still occurs within the rules of the game. All of the non-body checking league players had participated in a competitive body checking league prior to joining the non-body checking organization and we included them in this analysis because of their unique perspective of having viewed the issues from the point of view of both types of play. No other systematic differences could be inferred between groups; at the very least, however, no cohort effects could be attributed to factors such as age, as all players were between 13 and 14 years of age.

### Ethics

The Research Ethics Board of St. Michael's Hospital 03–315 (Q) has renewed their approval for this study (titled, for their purposes, "Clues to Prevention of Injury in Hockey: A Qualitative Study") for the years 2015–2016. Written informed consent was obtained from each player, parents/guardians, and the “reference others”.

### Data Collection

The basis for the interview outline was based on a review of the literature (peer reviewed journals and social psychology textbooks) and informal discussions with players, coaches, parents and health professionals. In-depth, semi-structured interviews were conducted with all participants. A full outline of the questions asked of participants is described in S1 Appendix. The average interview length was 38 minutes. Two pilot interviews were conducted with individuals not involved in the study, and feedback from those interviews helped to further develop the interview guide used. Upon completion of their interview, study participants were instructed not to disclose the content prior to the completion of all interviews. The voluntary nature of participation and assurances regarding privacy and confidentiality were emphasized before each interview.

### Data Analysis

All interviews were audiotaped, transcribed verbatim, and reviewed by members of the research team to identify major themes. Grounded theory methodology served generally as a framework, as categories and themes were allowed to emerge from the data inductively, as opposed to being pre-identified by a priori hypotheses [51, 52]. Text was divided into meaningful pieces of information known as meaning units (MU) coded based on similar features to create broad categories, and further classified into specific themes and sub-themes [53]. Only the themes pertaining to attitudes towards aggression in hockey are presented herein. The responses that aided in the identification of those themes were articulated in response to questions such as “Have you ever learned how to hit another player illegally in any way from watching pro-hockey?”, “What kind of things do you see when you watch hockey that bother you?”, and “How important is checking for winning in hockey?”

## Results

A thematic analysis of the interviews revealed themes related to aggression that were divided into four main categories: 1) players’ views on aggressive behaviors performed by others (see items *f* and *g* in Section One of S1 Appendix for examples), 2) parents’, coaches’ and teammates’ perceived influence on play (see items *e*, *f* and *g* in Section Three of S1 Appendix for examples), 3) players’ own aggressive thoughts and acts (see item *k* in Section Four of S1 Appendix, for example), and 4) players’ perceptions of parents’, coaches’, and teammates’ beliefs about aggression (see *l*, *m* and *n* in Section Four of S1 Appendix for examples), High injury rates have been observed in minor hockey, ranging from bone fractures to traumatic brain injuries [54–56]. In this analysis, we discuss how injuries to the head are associated with a high degree of emphasis on aggression within the sport. The themes we identified are described below.

### The players’ own aggressive behaviors

The players reported engaging in both instrumental and hostile aggression. Instrumental aggression is defined as legitimate action within the rules of the game, with the ultimate goal of advancing successful play. Conversely, the primary purpose of hostile aggression is to inflict harm on one’s opponent, often in cases where the player is angry [57].

Hostile aggression was exhibited by most of the competitive level players only as a response to a teammate being injured by an opponent. With female and non-body checking league players, anger was handled differently than in the body checking league. These players would talk about seeking revenge after a teammate was injured, but their feelings were not acted upon. One player described it in the context of major league incidents in the news where there had been severe injuries and felt that it was “right to revenge what happened to his fellow teammate, but not to that extent” and that to “stay out of the penalty box would help give their team an advantage…don’t take stupid penalties like slashing…There’s like a line where it’s ok and it’s not ok.” The desire to engage in revenge could be considered an outcome of the phenomenon known as groupthink, (the tendency of a group to make decisions in ways that discourage creative problem solving or individual responsibility) [58], and it is common to see this as a familiar tendency in sports teams [59]. However, it is noteworthy that there were few instances in these interviews where team members did act on such feelings.

There are, however, a variety of factors other than groupthink that may mediate action. For example research from sports in general (and around groupthink in particular) suggests that coaches, parents, and peers may be effective at demonstrating attitudes and behaviour that create a climate that reduces anxiety [60], there may be subtle interactions between a team’s overall sense of collective efficacy and their performance [61] or there may be changes in team membership or team dynamics that mitigate undue cohesion and risk of groupthink [62]. Moreover, such behaviour could also be because the coach’s attitude was not conducive to allowing retaliation, either because the players did not feel that acting on these feelings would be appropriate, or because of the rules of their game make physical acts of aggression illegal. In contrast, the competitive body checking-league players interviewed expressed a need to take matters into their own hands. For example, one player in our study observed that “it’s a physical game so you gotta hit to slow them down and stuff and it’s part of the game so…it’s pretty important like you have to do all of the stuff that makes you win and that’s one of them I think”.

Social identity theory provides some basis for understanding players’ feelings in this and similar scenarios, accounting for the strong feelings of responsibility on the part of group members to defend and protect each other in order to maintain the group’s cohesion [63, 64]. The relationship between participation in team sports and the development of prosocial behaviour and altruism is well-documented [65, 66], and recent research demonstrates a clear link between social identity and outcome interdependence (that is, the degree to which team performance is attributed to individual members’ performance and vice versa) [67].

A hockey team, like any small group, is going through a cycle of “form, storm, norm, perform, and adjourn” to form an effective team [68–70] where group cohesion serves to not only allow goals that are instrumental for the group to be pursued, but also to satisfy individual members’ affective needs [71, 72]. Similary, social conformity in sports is important as a team member may comply with an abusive or aggressive coach for the sake of social acceptance and approval. The need to maintain group cohesion through defending and protecting team mates may be contrasted with the fact that some players feel it is their role to initiate aggressive behaviors to intimidate the opponents, further intensified by their perception of their coach as emphasizing normative success and strongly wanting to win by capitalizing on “momentum”. As one player described it “if they’re scared, like if you get the momentum, that’s usually what happens, the coach wants us to get the momentum so we crank one guy and he kind of gets out of the play and starts shying off,” In such circumstances, they may perceive him or her as encouraging inappropriate aggression [73]. These players had higher self-reported numbers of penalty minutes than the team average.

The concept of designated roles, or even those that emerge informally is one that has been explored in the literature [74] and was revisited often throughout the interviews. Having a role on the team, even one designated as being an aggressive player, helps to secure one’s membership in the group, enhances group cohesion, and is encouraged by the players’ reference others. For example, one player identified the value of these roles thus: “making big hits and stuff would make you feel good and I guess scoring goals too. And anything to boost the team, so like energy I guess” while another noted that “if you’re big or small and you’re the guy who makes a big hit and makes the play that kind of picks up the team a bit so it helps if you have one guy who can do that”. This is also an illustration of masculinity and “being tough” linked with the players’ willingness to engage in aggressive behavior and to feel “manly” or “powerful” on the ice. This pressure still exists in the non-body checking league, but may be tempered by regulations that deter this behaviour.

There also appears to be a fundamental difference between males and females in their tolerance for aggression whether or not the male player was in the body-checking league or not. Although most of the female players interviewed said that they would be angry with a player who injured their teammate, they did not seek retribution. As one player stated, *“*At the time we get really mad and sometimes we’re like ‘oh let’s get her back’ or whatever, but usually we don’t.” This was evident not only with female players, but with female parents who were more likely to describe the game as extremely violent and to advocate for such regulation changes as stricter penalties for undue aggression. Although the male players intended to “hurt” another player in their pursuit of revenge, they did not believe that they were capable of seriously injuring their opponents, or were at least prone to minimize the impact of the violence. For example, male players in the body checking leagues made statements such as “the first year of hitting I hit somebody and I broke their collar bone, but like I guess I kind of did feel sorry for them and stuff, but not that guilty. I meant to hit them but I didn’t mean to put them out of the season.”, and “I’ve hit some people before, I don’t know, it was unintentional cause I didn’t expect what happened…like, I’ve hit some people and knocked them out, but I was mad at the time so I didn’t really mean to…. I just meant to show them I was there”. In other cases, they operationalized checking as just another tool to secure victory: *“*Checking yeah…it’s a big part of the game. You need to be physical to have a good team, so size matters a lot now, ‘cause if you’re small you’re not going to be able to check a lot. I think checking can help win a lot cause it can intimidate the other team if you’re a lot bigger than them, and you can sort of get the puck easier because they’ll be afraid of you checking them*”*.

Social theory states that men feel a need to exhibit aggressive behaviors through sports in order to develop their masculine identity [75], which may help to explain the variation in perspectives according to gender. This difference in gender perspectives is consistent across a variety of contexts [40, 76, 77]. This is an important issue to address when designing interventions, as raising the players’ awareness of the consequences of their actions may help to reduce this type of behaviour and its associated injuries.

### Players’ perceptions of their parents’, coaches’ and teammates’ views on aggression

According to Bronfenbrenner [78] parents, coaches and teammates represent people in immediate and proximal settings in which the individual (player) lives. This micro system includes the child’s or adolescent’s family, peer group, neighborhood and school life and helps shape a person's development through direct contact. The nature of this micro system is that the individuals who have direct contact with the young hockey player will aid in the construction of the settings of this system. Players’ perceptions of their parents, coaches and teammates are very important, as their feedback influences the development of their self-image and the corresponding behavior they exhibit on the ice [40, 41]. An interesting finding from our study is that players from the same team often had contrasting interpretations of their coaches’ and teammates’ beliefs. For example, one young player stated, regarding his coach, “He encourages all of us to do hitting, cause this one time he had a challenge for us to do 10 hits per period or 30 hits for the game and we ended up getting around 40-something…so he was happy cause we may have lost the game but we outplayed them just by hitting, and by the end of the first period they didn’t want to get hit anymore. So he knows that by encouraging people to hit, it will get us more into the game”, while another, from the same team, stated that “when we got into a fight he didn’t get too mad, he said that it wasted the end of the game, cause we could have won it”. This contrast among players’ experiences suggests, in the first example, a degree of groupthink at play, with a specific game culture self-evident to the participant; however, in the second quote considerably more ambiguity was reflected. Such contrasts could be accounted for by individuals’ relationships with the coaches and the team or due to their own values and beliefs, and their influence on their perceptions [33, 79].

Of the 11 players who were asked, the majority of them (7 when asked about their parents, 9 when asked about their coach, and 8 when asked about their teammates) stated that these reference others did not like illegal hits. Others described coaches who encouraged illegal hits in certain situations (mainly revenge-seeking). Parents were seen as being occasional advocates for illegal hits, so long as their child was not the initiator (“My dad sometimes says if the guy gives you a punch don’t take it, just give him a punch back.”). A negative influence on children’s behaviour in sport is not limited to hockey. In a US study of 132 junior tennis coaches it was found that coaches deemed parents to be a positive influence on their children (players) 59% of the time, but 36% of the time they perceived children’s behaviour during play to be negatively affected by parents (e.g., too much focus on winning, setting unrealistic goals, ongoing criticism of their child) [80]. Teammates were often seen as being occasional advocates for hitting, within proscribed limits. As one player described it, regarding his teammates’ behaviour, “Sometimes they just give a little pat on the back, like, you gotta be more aggressive out there and stuff…but nothing illegal”.

Being in the centre of the action, coaches are ideally placed to comment on sideline behaviour. Throughout the interviews, most participants clearly differentiated between legal and illegal hits, claiming that their reference others felt that checking was acceptable as long as the hit was “clean”. Clean hits refer to legal checks, while “cheap shots” refer to illegal hits, including hitting from behind, high sticking, and so forth. The two main reasons why reference others were said to express disapproval of illegal hits were that: 1) they were unfair and could result in injury and 2) that if their own players received penalties for illegal hits they might compromise the team’s chance of winning.

### Players’ views on others’ aggressive behaviour

The participants largely did not approve of illegal activities in professional hockey such as “high sticking”, and “cheap shots”. Although respondents disliked professionals behaving in this fashion, they felt it could be explained by the fact that the players felt frustrated or were “caught up in the heat of the moment”. As one player stated, regarding professionals indulging in unnecessary roughness, “They’re just so into the game that they forget what’s right and wrong.” In terms of attitudes towards their own team members, they were much less forgiving if it was seen as a cheap hit. As one player described it, “Well, if one of our players does a cheap hit, then we’ll care because we’d be disappointed in him, but if it’s a clean hit then we don’t care”.

Most of the female players and some of the male players reacted negatively when their teammates hit other players illegally, both because they felt that it was not appropriate and because they might get penalties and compromise the team’s chance of winning. Although it was seen as acceptable within the group to seek revenge, the goal of winning and maintaining a socially acceptable appearance was valued. As one young woman stated, “if someone hits someone else on the other team then we tell them they shouldn’t do that”. There was also a clear sense that verbal aggression, or “chirping” is seen as being a contributing factor to escalating anger and violence. As one player put it, “a lot of guys chirp…’cause they want to be tough and everything right but uh…I think it’s dumb…a lot of the time you see a guy, uh, like just being a pest and all that right and talking away or whatever. And the other guy will just like, uh, turn and slash him or elbow him or something and uh…yeah it’s dumb ‘cause then he just gets a penalty and the other guy, um…he just gets to go, right”.

### Parents’, coaches’ and teammates’ influence on youth play

Since both intrinsic and extrinsic factors affect a player’s behaviours [81], the influence of reference others on youth hockey players was explored. Our data showed that parents of players in the body checking league were more likely to accept checking as part of the game. In contrast, all of the parents of players in the non-body checking league as well as all of the female parents were more likely to be concerned about the risk of injury. Attitudes towards aggression between players and parents werecongruent as one of the non-body checking parents expressed it, “the kids [were] getting so much bigger than he was and it was just getting too dangerous for our liking. Like I said outside, he wanted to keep playing. He said ‘it’s just not worth the risk.’ We just didn’t want the chance…we saw other kids lying on the ice and you know, concussions and uh…we just thought you know, he can have fun somewhere else”

In contrast, many coaches emphasized legal means of aggression to avoid time in the penalty box, indicating the priority was the team’s ability to win the game. As one coach articulated it, “We’re building this team to make a good run through the playoffs as far as we can go, and um, you know, if you’re going to sit in the penalty box we’re not going to get there. We really preach a lot of discipline throughout the year and say, if you guys really want to be as good as you can be, you can’t be sitting in the penalty box”. Similarly, teammates mostly encouraged aggression, but in this case to obtain revenge when a teammate had been injured, consistent with previous themes regarding retaliation that emerged in this study. One of the body checking players put it most succinctly when he stated, “We try to injure them. Because if he injures one of our best players, you have to go after one of their best players to make it fair. Revenge is fair. It’s human nature. If someone does something to you, then you would want to do it back.”

Among reference others, tolerance for aggression within the game may also correlate with one’s exposure to hockey. Participants with a greater degree of involvement in organized hockey (i.e. Board members, coaches, parents who played hockey) were more likely to endorse aggression and physical violence in hockey and to perceive changes to body checking regulations as threatening. In contrast, parents who had no experience playing hockey appeared more supportive of changes to current regulations. All female parents strongly expressed the need for new policy to increase safety within the game. None of the female parents interviewed reported any personal hockey experience.

In summary, a number of factors might be seen to be related to an increase in aggression during hockey games, including a high emphasis on winning, revenge for teammates’ injuries and variances in the social/cultural values placed upon aggressive play.

## Discussion

While there have been other studies that have looked at shaping attitudes towards violence in hockey though education about sport-related concussions, addressed the role of culture (teammates, coaches, and parents) in determining when a child should return to play after a possible TBI [55, 82, 83], and explored, in a variety of sports, how general attitudes towards sport violence can be mediated [84–86] the study described herein is unique in its attempts to systematically explore the culture of hockey, and how that culture, through socialization, informs and influences young players’ attitudes towards violence in the course of the game. In general, the culture of hockey appears to encourage a sense of loyalty, which includes using aggression to defend teammates in order to protect the team’s cohesion. Warsh *et al*. found that leagues permitting body checking saw increased injuries attributable to body checking [15]. This relationship was also systematically explored by Emery *et al*. who found that Peewee players in leagues with body checking faced greater than a 3-fold risk of injury including severe concussion [6].

In the context of our findings, such aggression, and often the injuries that ensue, can often be attributable to impulsive, revenge-seeking behavior often modelled and encouraged by coaches, parents and teammates. This type of socialization is corroborated, encouraged and modelled also by media outlets through reporting sports violence and aggression in a light hearted manner under the auspice of sports entertainment. Together they account for socialization influences on young athletes’ developing notions of sports masculinity in a culture that promotes sports aggression and violence by deeming it entertaining. Players experience intense emotion on the ice, including anger, during which they disregard their injury-causing potential and perform an aggressive act “in the heat of the moment”. Both female players and those who have joined non-body checking leagues do not express anger in a physical sense; likewise, physical play is not reinforced by their reference others and is deterred by league regulations. In this way, our interpretation is consistent with findings by Emery et al that players in body checking leagues have a 2-fold increased risk of other intentional contact injuries, indicating they have a more aggressive style of play [6].

Rationalizing aggressive behavior enables the players to maintain the status of their sport, reinforcing their choice to participate in what some people may view as an uncivilized game because of the high rate of play-associated injuries. Most youth indicated that they did not respect professionals who acted like “goons”. This was reinforced by many participants who described their choice of role models as players who were smart, fast, and strong offensively rather than players known for their aggressive behaviors. Phrases such as “he’s really smart when he plays on the ice, he knows where everyone is when he’s playing” and “Crosby is just like really talented and stuff and he uses his head” are emblematic of this attitude. It is, perhaps, noteworthy that these attributes were most valued by non-body checking league players and female players.

### Potential Avenues for Prevention

The results of our qualitative assessment warrant the need for a greater understanding of the ways in which youth hockey socialization and young athletes’ notions of masculinity combine to create a culture of aggression and violence. Interventions must appeal to young players’ sense of competitiveness, while simultaneously developing their respect for and awareness of injury. The results of our study and those of others [4, 18, 87, 88] suggest that such interventions to curb aggression and injury in minor hockey should focus on educating youth players and their reference others, encouraging them to accept non-violent role models, and to be fully aware of the serious consequences for aggressive behaviour or head injury on the ice [89, 90].

In order to address these areas of change, multifaceted approaches are needed, targeting all levels of minor hockey, thereby addressing the needs of a population most in need of intervention. Ideally, these would include such steps as introducing universal rule changes to all levels of hockey and their strict, uniform enforcement, as well as broad educational and economic incentives and disincentives. For example, an increase in player and team play-related penalties as well as strict economic penalties and penalties that affect league standing to teams and leagues at all levels could quickly alter this culture of aggression. It is interesting to note that a review of 18 studies on the effectiveness of interventions to reduce aggression and injuries in minor hockey leagues [91] clearly illustrated that changes to mandatory rules were associated with both fewer penalties for aggressive acts and fewer aggression-related injuries, although the effects of educational and cognitive behavioural interventions were less clear. It was obvious from this review that well-designed studies of multifaceted strategies combining a number of approaches are required.

In terms of the relationship between exposure to hockey and tolerance for aggression, our findings support altering the makeup and renewal processes of governing bodies and governance structures. This could be accomplished by implementing time-limited terms of service for hockey organizations and increasing the presence of expert opinion in injury prevention. Such changes could promote relatively rapid change of hockey culture.

Physicians, health professionals, researchers, and concerned parents for their part, can help advocate for such interventions; serve as role models for a healthy approach to sport; counsel players, parents and coaches, and raise awareness about safe play and the risks associated with certain practices in this sport and other similar ones like rugby, American football and soccer.

### Limitations

The utility of qualitative data is strongly linked with the effectiveness of the researcher’s interviewing techniques. Although every attempt was made to keep interviews standardized and semi-structured not all interviews were conducted face-to-face. This may have introduced systematic variations in the type and detail of information shared.

Moreover, response bias in the expressed perspectives of those who chose to participate is a limitation of this study. Representativeness of the data in a qualitative study is important. Given the voluntary nature of the study, not all viewpoints may be represented. For example, coaches who refused to have their team participate in this study (and the team members as well as their parents) may have represented divergent positions which were not represented in the data. However, the range of expressed narratives obtained suggests indeed that the results are representative. While it might be argued that certain groups (i.e. only 2 managers; only 4 trainers) were not large enough on their own to attain saturation, it could be argued that the perspectives shared by the larger group of “reference others” did represent a large enough sample to allow for the emergence of meaningful themes. Certainly, given their key roles, these reference-others should be the focus of future research. To be certain, our sample size was large in comparison to many qualitative studies, but future work could try to gain greater representation of particular sets of reference others (i.e. game officials, female players).

## Conclusion

The dominant theme that emerged from our study showed that aggression is a part of the sport and participants justify it as a means to seek revenge, even if injury is a by-product of that aggression. Such revenge is not only seen as acceptable, but also reinforced by teammates, coaches, the media and the professional players whom the youth aspired to emulate, particularly in the context of competitive male body-checking leagues. With this new awareness about hockey culture, we encourage pursuing avenues to alter this dominant theme. Concerted efforts by all stakeholders inside and outside the sport using varied strategies will be required to achieve real change.

## Supporting Information

S1 Appendix (PDF)

## References

[1] Surveillance, Epidemiology Division CfCDPPHAoC. Chronic disease and injury indicator framework: quick stats, Fall 2014 edition. Chronic diseases and injuries in Canada. 2014;34(4):272–5. . 25408189

[2] Pediatrics AAo. Safety in youth ice hockey: the effects of body checking. Pediatrics. 2000;105(3 Pt 1):657–8. 10699128 10.1542/peds.105.3.657

[3] Bawa H BM, DeGagne D, Guanghong H, Smith D. Sports Related Injury Data Report 2001–2003: Emergency Department Injury Surveillance System: BC Research and Injury Prevention Unit; 2004. Available from: http://www.injuryresearch.bc.ca/wp-content/uploads/2014/08/EDISS-Sports-Related-Data-Report-2004.pdf.

[4] Brust JD, Leonard BJ, Pheley A, Roberts WO. Children's ice hockey injuries. Am J Dis Child. 1992;146(6):741–7. . 1595632 10.1001/archpedi.1992.02160180101026

[5] Emery CA, Hagel B, Decloe M, Carly M. Risk factors for injury and severe injury in youth ice hockey: a systematic review of the literature. Inj Prev. 2010;16(2):113–8. 10.1136/ip.2009.022764 . 20363818

[6] Emery CA, Kang J, Shrier I, Goulet C, Hagel BE, Benson BW, et al Risk of injury associated with body checking among youth ice hockey players. JAMA. 303(22):2265–72. Epub 2010/06/10. 303/22/2265 [pii] 10.1001/jama.2010.755 . 20530780

[7] Emery CA, Meeuwisse WH. Injury rates, risk factors, and mechanisms of injury in minor hockey. Am J Sports Med. 2006;34(12):1960–9. Epub 2006/07/25. 0363546506290061 [pii] 10.1177/0363546506290061 . 16861577

[8] Fu T, Jing R, Cusimano M. Lifetime costs of traumatic brain injury identified in the emergency department in Ontario. Canadian Journal of Neurological Sciences. 2015;42.

[9] Laker SR. Epidemiology of concussion and mild traumatic brain injury. PM R. 3(10 Suppl 2):S354–8. Epub 2011/11/09. S1934-1482(11)00498-9 [pii] 10.1016/j.pmrj.2011.07.017 . 22035677

[10] Marar M, McIlvain NM, Fields SK, Comstock RD. Epidemiology of concussions among United States high school athletes in 20 sports. Am J Sports Med. 40(4):747–55. Epub 2012/01/31. 0363546511435626 [pii] 10.1177/0363546511435626 . 22287642

[11] Marchie A, Cusimano MD. Bodychecking and concussions in ice hockey: Should our youth pay the price? CMAJ. 2003;169(2):124–8. Epub 2003/07/23. . 12874161 PMC164979

[12] Roberts WO. Hitting in amateur ice hockey: not worth the risk. The Physician and sportsmedicine. 1999;27(12):35–41. 10.3810/psm.1999.11.1102 . 20086683

[13] Shirazi M, Cusimano M, McFaull S. Violence-related brain injuries sustained in youth ice hockey. Canadian Journal of Neurological Sciences. 2015;42.

[14] Stuart MJ, Smith AM, Nieva JJ, Rock MG. Injuries in youth ice hockey: a pilot surveillance strategy. Mayo Clin Proc. 1995;70(4):350–6. . 7898141 10.4065/70.4.350

[15] Warsh JM, Constantin SA, Howard A, Macpherson A. A systematic review of the association between body checking and injury in youth ice hockey. Clin J Sport Med. 2009;19(2):134–44. 10.1097/JSM.0b013e3181987783 . 19451769

[16] Wojtys EM. Young Athletes. Sports Health: A Multidisciplinary Approach. 2015;7(2):108–9. 10.1177/1941738115572986 PMC4332650 25984254

[17] R M. The Bertuzzi Incident: On-Ice Attack Renews Debate About Violence in Hockey. Canada's Troubled Game Suffers Yet Another Blow. The Globe and Mail. 2004 3 10;Sect. A: 1.

[18] Bertuzzi's savage attack should land him in court. The Globe and Mail. 2004 3 10;Sect. A.

[19] Sports C. NHL Hockey Injuries: Canadian Broadcasting Corporation; 2015. Available from: http://sportsstats.cbc.ca/hockey/nhl-injuries.aspx?page=/data/nhl/injury/injuries.html%3f*hf=off*.

[20] Sheldon JP, Aimar CM. The role aggression plays in successful and unsuccessful ice hockey behaviors. Research Quarterly for Exercise and Sport. 2001;72(3):304–9. 11561397 10.1080/02701367.2001.10608965

[21] Almeida PL, Rubio VJ, Palou P, Olmedilla Zafra A. Psychology in the realm of sport injury. Revista de psicología del deporte. 2014;23(2):0395–400.

[22] Wiese-Bjornstal DM. Reflections on a Quarter-Century of Research in Sports Medicine Psychology. Revista de psicología del deporte. 2014;23(2):0411–421.

[23] Cusimano MD, Sharma B, Lawrence DW, Ilie G, Silverberg S, Jones R. Trends in North American newspaper reporting of brain injury in ice hockey. PLoS One. 2013;8(4):e61865 Epub 2013/04/25. 10.1371/journal.pone.0061865 PONE-D-12-40215 [pii]. . 23613957 PMC3629225

[24] Celozzi MJ, Kazelskis R, Gutsch KU. The relationship between viewing televised violence in ice hockey and subsequent levels of personal aggression. Journal of sport behavior. 1981.

[25] Nash JE, Lerner E. Learning from the pros: violence in youth hockey. Youth & Society. 1981;13(2):229–44.

[26] Villani S. Impact of media on children and adolescents: a 10-year review of the research. J Am Acad Child Adolesc Psychiatry. 2001;40(4):392–401. 10.1097/00004583-200104000-00007 . 11314564

[27] DeMatteo CA, Hanna SE, Mahoney WJ, Hollenberg RD, Scott LA, Law MC, et al My child doesn't have a brain injury, he only has a concussion. Pediatrics. 2010;125(2):327–34. 10.1542/peds.2008-2720 20083526

[28] Jeffery-Tosoni S, Fraser-Thomas J, Baker J. Parent Involvement in Canadian Youth Hockey: Experiences and Perspectives of Peewee Players. Journal of Sport Behavior. 2015;38(1):3–25.

[29] Gillis C. How parents and their lawyers are killing minor league hockey: Inside the madness that is driving kids, volunteers and referees out of Canada’s game. MacLean's. 2014.

[30] Bandura A. Social learning theory of aggression. J Commun. 1978;28(3):12–29. Epub 1978/01/01. . 690254 10.1111/j.1460-2466.1978.tb01621.x

[31] Merriam-Webster Online Dictionary. 2012. Culture.

[32] Emery CA, McKay CD, Campbell TS, Peters AN. Examining Attitudes Toward Body Checking, Levels of Emotional Empathy, and Levels of Aggression in Body Checking and Non-Body Checking Youth Hockey Leagues. Clinical Journal of Sport Medicine. 2009;19(3):207–15. 10.1097/JSM.0b013e31819d658e 00042752-200905000-00003. 19423973

[33] Kirker B, Tenenbaum G, Mattson J. An investigation of the dynamics of aggression: direct observations in ice hockey and basketball. Res Q Exerc Sport. 2000;71(4):373–86. Epub 2000/12/28. . 11125535 10.1080/02701367.2000.10608920

[34] Loughead TM, Leith LM. Hockey coaches' and players' perceptions of aggression and the aggressive behavior of players. Journal of Sport Behavior. 2001.

[35] Traclet A, Moret O, Ohl F, Clémence A. Moral disengagement in the legitimation and realization of aggressive behavior in soccer and ice hockey. Aggressive behavior. 2015;41(2):123–33. 25230671 10.1002/ab.21561

[36] Hancock B, Ockleford E, Windridge K. An introduction to qualitative research: Trent focus group Nottingham; 1998.

[37] Lacey A, Luff D. Trent Focus for research and development in primary health care: an introduction to qualitative analysis. Trent Focus Group. 2001.

[38] Patton MQ. Qualitative evaluation and research methods: SAGE Publications, inc; 1990.

[39] Taylor SJ, Bogdan R. A qualitative approach to the study of community adjustment. Monograph of the American Association of Mental Deficiency. 1981;(4):71–81. . 7231430

[40] Mugno DA, Feltz DL. The social learning of aggression in youth football in the United States. Can J Appl Sport Sci. 1985;10(1):26–35. Epub 1985/03/01. . 4006041

[41] Smith MD. Towards an explanation of hockey violence: A reference other approach. Canadian Journal of Sociology/Cahiers canadiens de sociologie. 1979:105–24.

[42] Field M, Collins MW, Lovell MR, Maroon J. Does age play a role in recovery from sports-related concussion? A comparison of high school and collegiate athletes. The Journal of pediatrics. 2003;142(5):546–53. 10.1067/mpd.2003.190 . 12756388

[43] Ilie G, Adlaf EM, Mann RE, Ialomiteanu A, Hamilton H, Rehm J, et al Associations between a history of traumatic brain injuries and current cigarette smoking, cannabis use, nonmedical opioid use, and elevated psychological distress in a population sample of Canadian adults. Journal of Neurotrauma. 2014;(1). Epub 2014 Dec 11. 10.1089/neu.2014.3619 PMC4504340 25496189

[44] Ilie G, Boak A, Adlaf EM, Asbridge M, Cusimano MD. Prevalence and correlates of traumatic brain injuries among adolescents. JAMA. 2013;309(24):2550–2. 10.1001/jama.2013.6750 . 23800930

[45] Ilie G, Mann RE, Boak A, Adlaf EM, Hamilton H, Asbridge M, et al Suicidality, bullying and other conduct and mental health correlates of traumatic brain injury in adolescents. PloS one. 2014;9(4):e94936 10.1371/journal.pone.0094936 24736613 PMC3988100

[46] Toledo E, Lebel A, Becerra L, Minster A, Linnman C, Maleki N, et al The young brain and concussion: imaging as a biomarker for diagnosis and prognosis. Neuroscience and biobehavioral reviews. 2012;36(6):1510–31. 10.1016/j.neubiorev.2012.03.007 22476089 PMC3372677

[47] Grindel SH. Epidemiology and pathophysiology of minor traumatic brain injury. Curr Sports Med Rep. 2003;2(1):18–23. 12831672 10.1249/00149619-200302000-00005

[48] Honey CR. Brain injury in ice hockey. Clin J Sport Med. 1998;8(1):43–6. . 9448957 10.1097/00042752-199801000-00010

[49] Juhn MS, Brolinson PG, Duffey T, Stockard A, Vangelos ZA, Emaus E, et al Position Statement. Violence and injury in ice hockey. Clinical journal of sport medicine: official journal of the Canadian Academy of Sport Medicine. 2002;12(1):46–51. . 11854591 10.1097/00042752-200201000-00014

[50] Marchie A, Cusimano MD. Bodychecking and concussions in ice hockey: Should our youth pay the price? Canadian Medical Association Journal. 2003;169(2):124–8. 12874161 PMC164979

[51] Rothe JP. Undertaking qualitative research: Concepts and cases in injury, health and social life: University of Alberta; 2000.

[52] Schwandt TA. The Sage dictionary of qualitative enquiry: Thousand Oaks, CA: Sage; 2007.

[53] Cohen J. Statistical power analysis for the behavioral sciences rev: Lawrence Erlbaum Associates, Inc; 1977.

[54] Cusimano MD, Taback NA, McFaull SR, Hodgins R, Bekele TM, Elfeki N. Effect of bodychecking on rate of injuries among minor hockey players. Open medicine. 2011;5(1):e57 22046222 PMC3205817

[55] Echlin PS, Tator CH, Cusimano MD, Cantu RC, Taunton JE, Upshur RE, et al A prospective study of physician-observed concussions during junior ice hockey: implications for incidence rates. Neurosurgical Focus. 2010;29(5):E4 10.3171/2010.9.FOCUS10186 21039138

[56] Macpherson A, Rothman L, Howard A. Body-checking rules and childhood injuries in ice hockey. Pediatrics. 2006;117(2):e143–e7. 16452323 10.1542/peds.2005-1163

[57] Freedman J, Carlsmith JM, Sears DO. Aggression Social Psychology. Toronto: Prentice-Hall of Canada; 1970 p. 101–32.

[58] Janis IL. Groupthink: University of Notre Dame Press; 1997.

[59] Hardy J, Eys MA, Carron AV. Exploring the potential disadvantages of high cohesion in sports teams. Small Group Research. 2005;36(2):166–87.

[60] Chapman J. Anxiety and defective decision making: an elaboration of the groupthink model. Management Decision. 2006;44(10):1391–404. 10.1108/00251740610715713

[61] Fuster-Parra P, García-Mas A, Ponseti F, Leo F. Team performance and collective efficacy in the dynamic psychology of competitive team: A Bayesian network analysis. Human movement science. 2015;40:98–118. 10.1016/j.humov.2014.12.005 25546263

[62] Beddoes-Jones F. The psychology of teams. Training Journal. 2004:16.

[63] Baumeister RF, Leary MR. The need to belong: desire for interpersonal attachments as a fundamental human motivation. Psychological bulletin. 1995;117(3):497–529. . 7777651

[64] Tajfel H, Turner JC. The Social Identity Theory of Intergroup Behavior. 2004.

[65] Kleiber DA, Roberts GC. The effects of sport experience in the development of social character: An exploratory investigation. Journal of Sport Psychology. 1981;3(2).

[66] Rutten EA, Stams GJJ, Biesta GJ, Schuengel C, Dirks E, Hoeksma JB. The contribution of organized youth sport to antisocial and prosocial behavior in adolescent athletes. Journal of Youth and Adolescence. 2007;36(3):255–64. 27519025 10.1007/s10964-006-9085-y

[67] Bruner MW, Eys M, Blair Evans M, Wilson K. Interdependence and Social Identity in Youth Sport Teams. Journal of Applied Sport Psychology. 2015;27(3):351–8. 10.1080/10413200.2015.1010661

[68] Anderson E. Sport, Theory and Socail Probles: A Critical Introduction. 1 ed New York: Routledge; 2010.

[69] Bruner MW, Eys, Mark A., Beauchamp, Mark R., Cote Jean. Examining the Origins of Team Building in Sport: A Citation Network and Genealogical Approach. Group Dynamics: Theory, Research, and Practice. 2013;17(1):30–42.

[70] Tuckman BW. Developmental Sequence in Small Groups. Psychological bulletin. 1965;63:384–99. . 14314073 10.1037/h0022100

[71] Carron AV. Cohesiveness in sport groups: Interpretations and considerations. Journal of Sport psychology. 1982.

[72] Chicau Borrego C, Cid L, Silva C. Relationship between group cohesion and anxiety in soccer. Journal of human kinetics. 2012;34(1):119–27. 23487008 10.2478/v10078-012-0071-z PMC3590828

[73] Bortoli L, Messina G, Zorba M, Robazza C. Contextual and individual influences on antisocial behaviour and psychobiosocial states of youth soccer players. Psychology of Sport & Exercise. 2012;13(4):397–406. 10.1016/j.psychsport.2012.01.001

[74] Cope CJ, Eys MA, Beauchamp MR, Schinke RJ, Bosselut G. Informal roles on sport teams. International Journal of Sport and Exercise Psychology. 2011;9(1):19–30.

[75] Kimmel M. The Life of a Man's Seasons: Male Identity in the Life Course of the Jock Changing Men. London: SAGE Publications Ltd; 1987 p. 53–68.

[76] Chalabaev A, Sarrazin P, Fontayne P, Boiché J, Clément-Guillotin C. The influence of sex stereotypes and gender roles on participation and performance in sport and exercise: Review and future directions. Psychology of Sport & Exercise. 2013;14(2):136–44. 10.1016/j.psychsport.2012.10.005

[77] Steinfeldt JA, Gilchrist G.A., Halterman A.W., Gomory A., Steinfeldt M.C. Drive for Muscularity and Conformity to Masculine Norms Among College Football Players. Psychology of Men & Masculinity. 12(4):324–38.

[78] Bronfenbrenner U. Interacting systems in human development. Research paradigms: Present and future. Persons in context: Developmental processes. 1988:25–49.

[79] Mageau GA, Vallerand RJ. The coach-athlete relationship: a motivational model. J Sports Sci. 2003;21(11):883–904. 10.1080/0264041031000140374 . 14626368

[80] Gould D, Lauer L, Rolo C, Jannes C, Pennisi N. Understanding the role parents play in tennis success: a national survey of junior tennis coaches. British journal of sports medicine. 2006;40(7):632–6. 16702176 10.1136/bjsm.2005.024927 PMC2564313

[81] Fuster-Parra P, García-Mas A, Ponseti F, Palou P, Cruz J. A Bayesian network to discover relationships between negative features in sport: a case study of teen players. Quality & Quantity. 2014;48(3):1473–91.

[82] Echlin PS. Editorial: Concussion education, identification, and treatment within a prospective study of physician-observed junior ice hockey concussions: social context of this scientific intervention. Neurosurgical focus. 2010;29(5):E7. 10.3171/2010.10.FOCUS10222 21039141

[83] Echlin PS, Johnson AM, Holmes JD, Tichenoff A, Gray S, Gatavackas H, et al The Sport Concussion Education Project. A brief report on an educational initiative: from concept to curriculum: Special article. Journal of neurosurgery. 2014;121(6):1331–6. 10.3171/2014.8.JNS132804 25280091 PMC4386878

[84] Ford DW, Jubenville CB, Phillips MB. The Effect of the Star Sportsmanship Education Module on Parents' Self-Perceived Sportsmanship Behaviors in Youth Sport. Journal of Sport Administration and Supervision. 2012;4(1).

[85] Frederick EL, Lim C. H., Chung J. & Clavio G. Determining the Effects of Sport Commentary on Viewer Perceptions, Attitudes, Beliefs, and Enjoyment through Violence Justification. 2013 Contract No.: 1.

[86] Perry JL, Clough PJ, Crust L. Psychological approaches to enhancing fair play. Athletic Insight. 2013;5(2):197.

[87] Arnold E. Whose puck is it anyway? A season with a minor novice hockey team. Toronto: McClelland & Steward; 2002.

[88] Marcotte G, Simard D. Fair-play: an approach to hockey for the 1990s. ASTM SPECIAL TECHNICAL PUBLICATION. 1993;1212:103-.

[89] Demorest RA, Landry GL. Prevention of pediatric sports injuries. Curr Sports Med Rep. 2003;2(6):337–43. . 14583164 10.1249/00149619-200312000-00010

[90] Shah CP. Public health and preventive medicine in Canada: WB Saunders Company Canada Limited; 2003.

[91] Cusimano MD, Nastis S, Zuccaro L. Effectiveness of interventions to reduce aggression and injuries among ice hockey players: a systematic review. Canadian Medical Association Journal. 2012:cmaj. 112017. 10.1503/cmaj.112017 PMC3537813 23209118

