# Supplementary material for: Aggression, Violence and Injury in Minor League Ice Hockey: Avenues for Prevention of Injury
Source: PLoS One. 2016 Jun 3;11(6):e0156683. doi: 10.1371/journal.pone.0156683 (PMC4892613; doi:10.1371/journal.pone.0156683)
Supplement: S1 Appendix — (PDF) [file pone.0156683.s001.pdf]

Appendix 1: GUIDELINES FOR INTERVIEW QUESTIONS

This section sees the addition of seven new questions to be asked during the semi-structured interview (outlined in bold font). Current evidence supports why these new questions can help to better address young players' attitudes towards aggression and injuries in hockey.

Introduction to players: Hi, my name is Sarah. We are going to talk about hockey. I want you to keep in mind that this is not a test and that you should try and answer with how you really feel and not with what you think you should answer. I also want you to know that I am not going to talk to your parents or coaches about your answers. If there are any questions that you don't feel comfortable answering then you don't have to. I will be documenting your responses by taking notes as well as with this audiotape. Are you ready to begin?

Section 1: Mass Media Portrayals

- Who is your favourite hockey player? Why?
- If you had to describe that player in three words what would they be? Do you read about hockey in the newspaper, in magazines, or on the internet? How often?
- Do you watch stuff about hockey on TV or hear about it on the radio?
- How many NHL games do you watch a week?
- Have you ever tried to imitate a pro in your games? In what sort of ways?
- Have you ever learned how to hit another player illegally in any way from watching pro-hockey?
- What kind of things do you see when you watch hockey that bother you?
- What should not be done by NHL players? Do they still do it? Why?
- Do you think that this is right?
- What kind of things should NHL players do to make them win more?
- What kind of things should NHL players do to get fewer injuries?
- **What is your favourite part of watching an NHL game?**

Section 2: Social Organization of Hockey

- Why do you play hockey?
- What is the most important reason that you play hockey?
- Do you feel that winning your games is important? Why?
- How important is checking for winning in hockey?
- **What qualities/characteristics of a person help to make them a good hockey player? Which do not help make a hockey player?**
- Do you want to advance to a higher level of play i.e. AA/AAA or even get a hockey scholarship?
- If so, what kind of things do you need to do to advance? Why do you think that?

- **What type of players do you think are most likely to get injured in hockey?**

Section 3: The influence of players' reference others (teammates, parents, coaches)

- Do you feel any pressure to win your games? From whom?
- Do you only have fun if you win?
- Do you feel any pressure to improve your game or advance to a higher level of play?
- **What are some of the unofficial roles of players on your team in addition to their position on the ice?**
- Does your coach encourage you to be rough/physical? Describe how. Why?
- Do your parents encourage you to be rough/physical? Describe how. Why?
- Do your teammates encourage you to be rough/physical? Describe how. Why?
- How does your coach determine the amount of ice time each player gets?
- How do you and your friends react when a player from the other team injures one of your teammates? Why?
- How do you and your friends react when someone from the other team gets injured? Why?

Section 4: Attitudes on Safety

- Do you think that safety is more important than winning/shifts/fun in hockey?
- What is the most serious kind of injury? Why?
- Do you think that you could get injured in this way?
- Do you think that hitting from behind is dangerous?
- Do you think you could seriously injure someone this way?
- Did you ever see this happen? When?
- Has anyone on your team ever been "knocked out"?
- What did you and your teammates feel about that?
- **What are some of the possible consequences of getting injured? What do you think is the worst thing that could happen to you from an injury?**
- Have you ever been injured in a hockey game? What happened? What was your injury?
- Have you ever injured another player in a hockey game? What happened? How did it make you feel? How do you think the player who got hurt felt? What was the effect on the game? What did your parents/coach say?
- Does your coach feel that safety is more important than winning in hockey?*
- What has he/she done in the past to promote safe behavior?*
- How does your coach feel about illegal hits in your games? You team giving them? Your team getting them?*
- Which does your coach feel is more important: having fun or winning?*
- If someone wanted to teach you about safety do you think that you would be interested in learning about it?
- Do you think that learning about safety would change the way you play hockey? If so,

would you learn best from a video, your parents, or your coach? How about some other way?

Do you have any questions about the project that you would like to ask?

* Ask these same questions about their parents and teammates

Additions to Interview Guideline:

Section 1: Mass Media Portrayals

With regard to assessing how the media influences young adolescents' attitudes towards aggression in hockey, we plan to ask "*What is your favourite part of watching an NHL game*?". It is well-documented that youth are influenced by violence in the media through positive reinforcement of violent behaviours and desensitization to the consequences of violence. (DuRant et al., 2008). If youths positively describe violent events while watching hockey on television (i.e. fighting between players), it is hypothesized that they are more likely to engage in these behaviours, putting themselves and others at greater risk for injury.

Section 2: Social Organization of Hockey

In this section, we plan to add three interview questions to help better address our research question. By asking, "*What qualities/characteristics of a person help to make them a good hockey player? Which do not help make a good hockey player*?", we hope to elicit whether characteristics such as aggressive tendencies are viewed as beneficial in hockey. Smith (1988) argues that violence is encouraged in hockey culture and is believed by coaches, parents, and teammates to demonstrate strong character. He indicates that players' perceptions of this attitude affect their on-ice behaviour and may become more evident with greater exposure. Additionally, by asking, "*What type of players are most likely to get injured in hockey*?" we can gain further insight into the players' attitudes regarding injury susceptibility. It has been found that characteristics such as small physical size, poor endurance and lack of preseason training are associated with increased injury rates in youth hockey players (Emery, 2003, Emery et al., 2009).

Section 3: The influence of players' reference to others (teammates, parents, coach)

It has been found that intimidation by violence is a common factor in youth hockey and is associated with activities such as high-sticking, fighting and illegal checking. This type of behaviour may be reinforced by teammates, parents and coaches, and has resulted in many teams having an "enforcer" on their roster to intimidate the opposition through violence. (Juhn et al., 2002). By additionally asking, "*What are some of the unofficial roles of players on your team in*

addition to their position on the ice?", we hope to determine whether the team encourages such unofficial positions as an enforcer to promote intimidation through violence.

Section 4: Attitudes on Safety

Upon questioning participants about their attitudes on safety, we plan to ask "*What are some of the possible consequences of getting injured? What do you think is the worst thing that could happen to you from an injury*?" Injuries affecting the head, neck and face regions make up 20 – 30% of all injuries in youth hockey with concussions being the most common sports-related injury. (Moslener & Wadsworth, 2010). There is significant under-reporting of concussions as many do not involve a loss of consciousness and players are concerned that they will be removed from practice or games. Youth hockey players in particular need to be aware that concussions can have detrimental long-term consequences, especially if they occur repetitively. (Juhn et al., 2002; Williamson & Goodman, 2006.). Through this question, we hope to gain a better understanding of players' knowledge regarding susceptibility to injury and the possible consequences of sustaining injury from the game.
